# Endothelial-Enriched lncRNA Gm39822 Modulates Inflammation and Dysfunction in Non-Diabetic Endothelial Cells

**DOI:** 10.3390/ijms26178147

**Published:** 2025-08-22

**Authors:** Amit Chandra, Emre Bektik, Vinay Randhawa, Mark W. Feinberg

**Affiliations:** Cardiovascular Division, Department of Medicine, Brigham and Women’s Hospital, Harvard Medical School, Boston, MA 02115, USA; achandra6@bwh.harvard.edu (A.C.);

**Keywords:** endothelial dysfunction, lncRNA, diabetes, inflammation

## Abstract

Endothelial dysfunction underlies several vascular complications, including diabetes and atherosclerosis. However, the underlying role of long non-coding RNAs (lncRNAs) remains poorly understood. This study elucidated the role of lncRNA Gm39822 in regulating endothelial dysfunction under healthy and diabetic conditions. Our data revealed that Gm39822 is enriched and upregulated in non-diabetic endothelial cells when exposed to high glucose or inflammatory cytokines (TNF-α and IL-1β). Gm39822 overexpression promoted the expression of vascular cell adhesion molecule-1 (VCAM-1) and the adhesion of leukocytes in non-diabetic ECs but not in diabetic ECs. Conversely, Gm39822 silencing reduced VCAM1 expression and leukocyte adhesion in non-diabetic ECs and not in diabetic ECs. Gm39822 deficiency reduced the expression of inflammatory mediators (including p-P65, P65, P50, p-P38, P38, P-ERK1/2, and ERK1/2) in non-diabetic ECs. Furthermore, Gm39822 knockdown inhibited the secretion of pro-inflammatory cytokines, including TNF-α, IL-1β, and IL-6, suggesting that Gm39822 regulates EC inflammatory responses. Mechanistically, we identified C1D, a nuclear-enriched corepressor, as an interacting partner of Gm39822 that could play an important role in mediating Gm39822 functions in non-diabetic ECs. Collectively, our results identify a novel lncRNA Gm39822 and provide insights into the molecular mechanisms underlying endothelial dysfunction. These findings highlight Gm39822 as a potential therapeutic target for mitigating vascular complications associated with non-diabetic endothelial dysfunction.

## 1. Introduction

Endothelial dysfunction underlies the onset and progression of several cardiovascular diseases, including atherosclerosis, hypertension, and diabetes mellitus. Endothelial homeostasis can be influenced by various external and internal cues, such as inflammation, dyslipidemia, and obesity, that may lead to increased vascular permeability, oxidative stress, and impaired vasodilation [1]. Despite optimized medication therapy and lifestyle modifications, endothelial dysfunction can persist, and the underlying etiologies are poorly understood.

Recently, long non-coding RNAs (lncRNAs) have emerged as crucial regulatory molecules in health and disease, including cardiovascular disease. LncRNAs are non-coding transcripts greater than 200 nucleotides in length that can interact with DNA, other non-coding RNAs (e.g., microRNAs), mRNAs, or proteins to regulate gene expression by various mechanisms, including chromatin remodeling, transcriptional, translational, and post-transcriptional regulations [2]. The human genome encodes thousands of lncRNAs, many of which are evolutionarily poorly conserved yet exhibit a cell-type- or stimulus-specific expression pattern and are localized to a particular subcellular fraction [3]. A growing body of evidence now highlights the role of lncRNAs in maintaining endothelial homeostasis in vascular inflammation and atherogenesis. For example, lncRNAs, MALAT1, ANRIL, SENCR, SNHG12, and VINAS have been shown to regulate different aspects of endothelial proliferation, migration, inflammatory responses, or vascular senescence [4,5,6,7,8]. These findings underscore the importance of lncRNAs in cardiovascular pathology and suggest that identifying novel lncRNAs with endothelial regulatory functions could provide valuable insights into disease mechanisms.

In this study, we identified the lncRNA Gm39822, a previously uncharacterized lncRNA, as a critical regulator of endothelial cell functions. Our findings reveal that Gm39822 exerts distinct and contrasting effects under diabetic and non-diabetic conditions. Gm39822 acts as a pro-inflammatory lncRNA in non-diabetic mouse endothelial cells (mECs). Interestingly, Gm39822 exhibits an anti-inflammatory phenotype in diabetic endothelial cells (db mECs). This context-dependent role of Gm39822 suggests a dynamic regulatory mechanism influenced by the metabolic status and disease conditions. Further mechanistic analysis revealed that Gm39822 interacts with C1D, a nuclear-enriched DNA-binding corepressor, specifically in non-diabetic mECs and not diabetic mECs.

Given the growing interest in lncRNAs as therapeutic targets, understanding the molecular mechanisms underlying Gm39822 function may provide new strategies for modulating endothelial inflammation and vascular dysfunction in cardiovascular diseases. This study highlights the complex role of Gm39822 in endothelial physiology and underscores the contrasting roles of a lncRNA in context-specific conditions.

## 2. Results

### 2.1. Gm39822 Is an Endothelial-Enriched lncRNA Localized in the Nucleus

We have previously identified non-coding RNAs that play key roles in diabetes-associated atherosclerosis [9,10]. To investigate changes in the aortic transcriptome associated with this condition, we used LDL-receptor-deficient (LDLR^–/–^) male mice fed on a high-fat sucrose diet (HFSC) for up to 12 weeks to simulate atherosclerosis progression. Aortic intima were isolated from HFSC-diet-fed mice at 0, 2, and 12 weeks, and bulk RNA sequencing was performed; 140 lncRNAs were differentially expressed at the 12-week time point, as reported previously [9]. In this study, we sought to identify potential endothelial-enriched lncRNAs from this set of lncRNAs that could play a key role in the physiology of endothelial cell function. Herein, we report Gm39822, an endothelial-enriched lncRNA, which exhibits a context-specific role in maintaining endothelial function.

**Figure 1:**
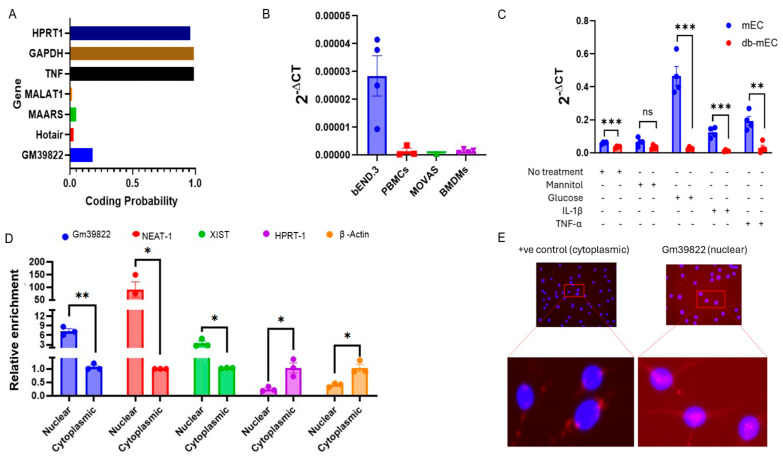
Gm39822 is an endothelial-enriched long non-coding RNA localized to the nucleus. (**A**) Coding potential analysis of Gm39822 using coding potential assessment tool (CPAT) showing the low coding potential of the lncRNA Gm39822. (**B**) Expression of the lncRNA Gm39822 in various vascular cell types, including brain endothelial cells (bEND.3), peripheral blood mononuclear cells (PBMCs), MOVAS, and bone-marrow-derived macrophages (BMDMs) (*n* = 4/group). (**C**) Expression of the lncRNA Gm39822 in non-diabetic (mECs) and diabetic (db mECs) endothelial cells under no treatment, mannitol (25 mM), glucose (25 mM), IL-1β (10 ng/mL), and TNF-a treatment (10 ng/mL) (*n* = 4/group). (**D**,**E**) Expression of the lncRNA Gm39822 in the cytoplasmic and nuclear fractions of bEND3 cells, normalized to the cytoplasmic fraction (*n* = 3/group) as determined by (**D**) qPCR and (**E**) RNAScope. The data are represented as the mean ± SEM, and statistical significance was determined with an unpaired two-tailed Student *t*-test. ns—non significant, * *p* < 0.05, ** *p* < 0.01, *** *p* < 0.001.

The long non-coding RNA Gm39822 encompasses a length of 2.284 kb in the forward strand of chromosome 11 (from 51,789,793 to 51,800,451 nucleotides) in the mouse genome. Gm39822 is predicted to be a non-coding RNA, as is also observed by its low coding potential score (0.18), which closely resembles the scores of previously identified non-coding RNAs, such as MALAT1 (0.02), MAARS (0.05), and HOTAIR (0.03), compared to the score of protein coding genes, such as HPRT1 (0.96), GAPDH(0.99), and TNF (0.99) (Figure 1A). Further quantification of Gm39822 via qRT-PCR showed that it is particularly enriched in mouse brain endothelial cells (bEND.3) when compared to PBMCs, MOVAS (mouse aortic smooth muscle cells), and BMDMs (bone-marrow-derived macrophages (Figure 1B). Furthermore, we observed that Gm39822 is more abundant in endothelial cells derived from non-diabetic mice (mEC) than the diabetic mouse ECs (db mEC) (Figure 1C). Interestingly, treatment of high glucose or inflammatory stimuli (TNF-α, or IL-1β) induced the expression of Gm39822 in the mECs, but there was no change in the Gm39822 expression in the db mECs after treatment with either glucose or inflammatory stimuli (TNF-α, or IL-1β) (Figure 1C). The subcellular localization of lncRNAs can be a primary indicator of their potential functions, such as regulation of chromatin or transcription in the nucleus or translational regulation in the cytoplasm [11]. Therefore, we quantified the relative abundance of Gm39822 in the nuclear and cytosolic fractions of total RNAs in the bEND.3 cells. The qRT-PCR data showed that Gm39822 is significantly enriched in the nuclear fraction compared to the cytosolic fraction (Figure 1D). In this experiment, we used previously identified nuclear lncRNAs (XIST and NEAT1) as positive controls for the nuclear fraction and mRNA of HPRT1 and β-actin as positive controls for the cytosolic fraction of total RNA. We further validated that Gm39822 is nuclear-enriched in bEND.3 cells by RNA-ISH confirming that Gm39822 is enriched in the nucleus (Figure 1E).

### 2.2. Gm39822 Promotes the Expression of VCAM-1 and Attachment of Monocytes to Non-Diabetic Endothelial Cells but Not in Diabetic Endothelial Cells

**Figure 2:**
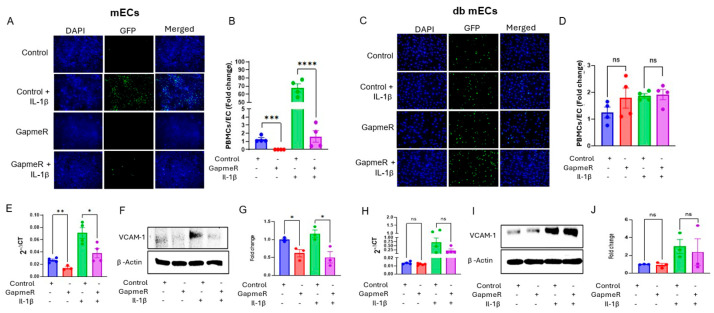
Inhibition of Gm39822 suppresses the adhesion of leukocytes to endothelial monolayers in primary non-diabetic endothelial cells (mECs) but not in diabetic mECs (Db-mECs). (**A**,**B**) Silencing of Gm39822 reduces the adhesion of PBMCs to non-diabetic endothelial cells (mECs) under basal conditions and 8 h of IL-1β stimulation (20 ng/mL, *n* = 4/group). (**C**,**D**) Silencing of Gm39822 does not impact the adhesion of PBMCs to diabetic endothelial cells (db mECs) under either basal conditions or 8 h of IL-1β stimulation (20 ng/mL, *n* = 4/group). (**E**) Inhibition of Gm39822 downregulates the expression of VCAM-1 at the mRNA level in non-diabetic endothelial cells (mECs) under basal conditions and IL-1β stimulation (10 ng/mL for 4 h, *n* = 4/group). (**F**,**G**) Inhibition of Gm39822 downregulates the expression of VCAM-1 protein in non-diabetic endothelial cells (mECs) under basal conditions and IL-1β stimulation (20 ng/mL for 8 h, *n* = 3/group). (**H**) Inhibition of Gm39822 does not impact the expression of VCAM-1 at the mRNA level in diabetic endothelial cells (db mECs) under either basal conditions or IL-1β stimulation (10 ng/mL for 4 h, *n* = 4/group). (**I**,**J**) Inhibition of Gm39822 does not impact the expression of VCAM-1 protein in diabetic endothelial cells (db mECs) under either basal conditions or IL-1β stimulation (20 ng/mL for 8 h, *n* = 3/group). The data are represented as the mean ± SEM, and statistical significance was determined with an unpaired two-tailed Student *t*-test. ns—non significant, * *p* < 0.05, ** *p* < 0.01, *** *p* < 0.001, **** *p* < 0.0001.

We next sought to decipher the functional roles of Gm39822 in endothelial cells under basal and pro-inflammatory stimuli using knockdown and overexpression strategies in bEND.3 cells, mECs, and db-mECs (Supplementary Figure S1A–F). Because pathological activation of ECs leads to excessive recruitment of leukocytes to the vascular endothelium and is a key event in atherosclerosis progression [12], we quantified the impact of Gm39822 on the expression of VCAM-1 (vascular cell adhesion molecule-1) and monocyte recruitment to ECs. The GapmeR-mediated knockdown of Gm39822 decreased, whereas overexpression of Gm39822 increased leukocyte adhesion to bEND.3 cells (Supplementary Figure S2A–C). In line with this, Gm39822 knockdown downregulated VCAM-1 expression, whereas overexpression of Gm39822 upregulated VCAM-1 expression at the mRNA (Supplementary Figure S2D,E) and protein levels (Supplementary Figure S2F–I), under both basal and TNF-α treatment. Subsequently, we extended our study to primary mECs and db mECs. Similar to observations in bEND.3 cells, GapmeR-mediated knockdown of Gm39822 decreased the number of monocytes attached to mECs under both basal and IL-1β-stimulated conditions (Figure 2A,B). Interestingly, the number of monocytes attached to db mECs did not change due to Gm39822 knockdown under either the basal or IL-1β treatment (Figure 2C,D). VCAM-1 is a prominent membrane protein that mediates the attachment of leukocytes to ECs and consequently plays a key role in the development of atherosclerosis. Therefore, we quantified the expression of VCAM-1 post-Gm39822 knockdown and overexpression in mECs and db ECs. We found that knockdown of Gm39822 inhibited the expression of VCAM-1 at both the RNA (Figure 2E) and protein levels (Figure 2F,G) in mECs but not in db-mECs (Figure 2H–J).

**Figure 3:**
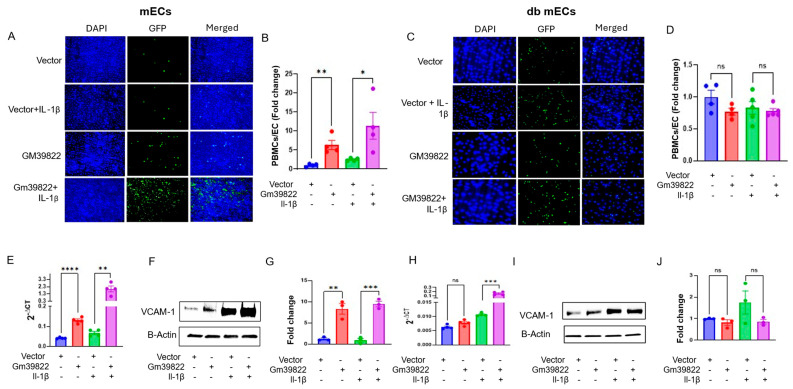
Overexpression of Gm39822 promotes the adhesion of leukocytes to endothelial monolayers in mECs but not in db mECs. (**A**,**B**) Overexpression of Gm39822 increases the adhesion of PBMCs to non-diabetic endothelial cells (mECs) under basal conditions and 8 h of IL-1β stimulation (20 ng/mL, *n* = 4/group). (**C**,**D**) Overexpression of Gm39822 does not impact the adhesion of PBMCs to diabetic endothelial cells (db mECs) under either basal conditions or 8 h of IL-1β stimulation (20 ng/mL, *n* = 4/group). (**E**) Overexpression of Gm39822 upregulates VCAM-1 expression at the mRNA level in non-diabetic endothelial cells (mECs) under basal conditions and IL-1β stimulation (10 ng/mL for 4 h, *n* = 4/group). (**F**,**G**) Overexpression of Gm39822 upregulates the expression of VCAM-1 at the protein level in non-diabetic endothelial cells (mECs) under basal conditions and IL-1β stimulation (20 ng/mL for 8 h, *n* = 3/group). (**H**) Overexpression of Gm39822 does not impact VCAM-1 expression at the mRNA level in diabetic endothelial cells (db mECs) under either basal conditions or IL-1β stimulation (10 ng/mL for 4 h, *n* = 4/group). (**I**,**J**) Inhibition of Gm39822 does not impact the expression of VCAM-1 protein in diabetic endothelial cells (db mECs) under either basal conditions or IL-1β stimulation (20 ng/mL for 8 h, *n* = 3/group). The data are represented as the mean ± SEM, and statistical significance was determined with an unpaired two-tailed Student *t*-test. ns—non significant, * *p* < 0.05, ** *p* < 0.01, *** *p* < 0.001, **** *p* < 0.0001.

In line with these findings, the overexpression of Gm39822 in mECs increased the attachment of leukocytes to mECs (Figure 3A,B) but not to db mECs (Figure 3C,D). As expected, Gm39822 overexpression induced VCAM-1 expression at the mRNA (Figure 3E) and protein levels (Figure 3F,G) in mECs, whereas there were no changes in VCAM-1 expression following Gm39822 overexpression in db mECs at either the mRNA (Figure 3H) or protein levels (Figure 3I,J).

### 2.3. Gm39822 Regulates the Transcriptomic Network of Cytokine Production in Endothelial Cells

**Figure 4:**
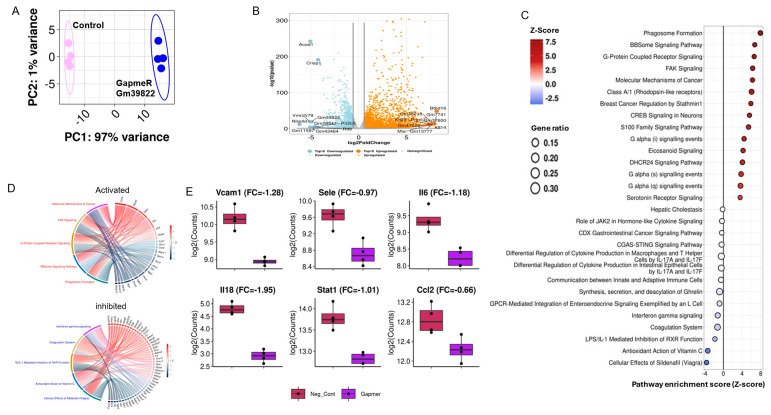
IPA canonical pathway analysis after Gm39822 knockdown in ECs reveals downregulation of cytokine production. (**A**) Principal component analysis (PCA) of variance-stabilized counts, with PC1/PC2 percentages of explained variance and 95% confidence ellipses. (*n* = 4). (**B**) Volcano plots of differential expression (*p* < 0.01 and |log_2_FC| ≥ 0.58) in GapmeR versus negative-control-treated bEND.3 cells. Up- and downregulated transcripts are colored red/blue; the top 10 transcripts in each class are labeled with a gene symbol. (*n* = 4). (**C**) IPA canonical pathway analysis and dot plot of all canonical pathways with significant activation (z-score > 0) or inhibition (z-score < 0) (*p* < 0.05) after Gm39822 knockdown compared with the control. Dot size = gene ratio; color gradient = z-score; pathways are sorted by the absolute z-score within each class (*n* = 4). (**D**) GO chord plots showing the significantly regulated genes (z-score < 0, *p* < 0.05) involved in the top 5 activated (top) or inhibited (bottom) pathways when comparing Gm39822 knockdown with controls (*n* = 4). (**E**) Box plots of normalized expression for selected marker genes in the “cytokine production” pathway, namely, Vcam-1, E-Selectin (SelE), Il-6, Il-18, Stat-1, and ccl2, in GapmeR versus negative control samples (*n* = 4). The inset in each panel shows the log2FC (FC) from the DESeq2 Wald test.

To further explore the molecular pathways underlying the functional role of Gm39822, we performed RNA sequencing after Gm39822 knockdown in bEND.3 cells. Principal component analysis showed good separation of control and Gm39822 knockdown samples (Figure 4A). Gm39822 knockdown altered the expression of 4607 genes, of which 2568 genes were upregulated, and 2039 genes were downregulated at an adjusted *p*-value of <0.05 (Figure 4B). Pathway analysis of downregulated genes showed “cytokine production in intestinal epithelial cells” and “interferon gamma signaling” among the top downregulated pathways, whereas “phagosome formation” was the top upregulated pathway (Figure 4C). GO chord plots of the top downregulated and upregulated (Figure 4D) pathways revealed several known transcripts associated with these pathways. As expected, the knockdown of Gm39822 decreased the transcript levels of VCAM-1 (Figure 4E). We assessed for specific transcripts within cytokine production that were downregulated and found several proinflammatory markers that were suppressed, including SELE, IL6, IL18, STAT1, and CCL2 (Figure 4E). These findings suggest that Gm39822 may regulate inflammatory pathways.

### 2.4. Knockdown of Gm39822 Suppresses Multiple Pathways of Inflammation in Non-Diabetic Endothelial Cells but Promotes Inflammatory Pathways in Diabetic Endothelial Cells

**Figure 5:**
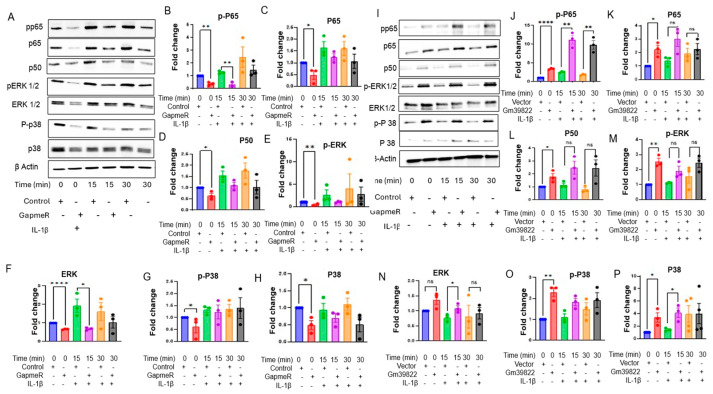
Gm39822 is a proinflammatory lncRNA in non-diabetic endothelial cells (mECs). (**A**–**H**) The expression of inflammatory signaling pathways as detected using Western blot analysis (**A**) for (**B**) p-p65, (**C**) p65, (**D**) p50, (**E**) ERK1/2, (**F**) p-ERK1/2, (**G**) p-p38, and (**H**) p38 expression after knockdown of Gm39822 in non-diabetic endothelial cells (mECs) under basal conditions or Il-1β stimulation (20 ng/mL; *n* = 3/group) at the indicated time points. (**I**–**P**) The expression of inflammatory signaling pathways as detected using Western blot analysis (**I**) for (**J**) p-p65, (**K**) p65, (**L**) p50, (**M**) p-ERK1/2, (**N**) ERK1/2, (**O**) p-p38, and (**P**) p38 expression after Gm39822 overexpression in non-diabetic endothelial cells (mECs) under basal conditions or Il-1β stimulation (20 ng/mL; *n* = 3/group) at the indicated time points. The data are represented as the mean ± SEM, and statistical significance was determined with an unpaired two-tailed Student *t*-test. ns—non significant,* *p* < 0.05, ** *p* < 0.01, **** *p* < 0.0001.

**Figure 6:**
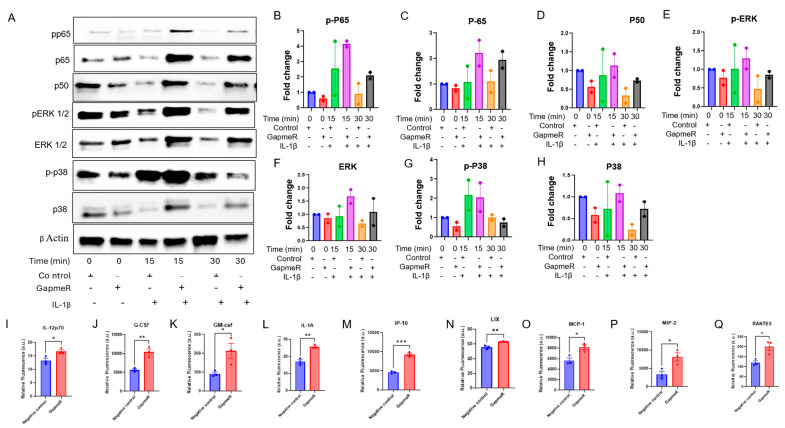
Gm39822 is an anti-inflammatory lncRNA in non-diabetic endothelial cells (db mECs). (**A**–**H**) The expression of inflammatory signaling pathways as detected using Western blot analysis (**A**) for (**B**) p-p65, (**C**) p65, (**D**) p50, (**E**) p-ERK1/2, (**F**) ERK1/2, (**G**) p-p38, and (**H**) p38 expression after knockdown of Gm39822 in diabetic endothelial cells (mECs) under basal conditions or Il-1β stimulation (20 ng/mL; *n* = 2/group) at the indicated time points. Quantification of cytokines (**I**) IL-12p70, (**J**) G-CSF, (**K**) GM-CSF, (**L**) IL-1A, (**M**) IP-10, (**N**) LIX, (**O**) MCP-1, (**P**) MIP-2, and (**Q**) RANTES in the supernatants from diabetic ECs after Gm39822 knockdown, *n* = 3/group. The data are represented as the mean ± SEM, and statistical significance was determined with an unpaired two-tailed Student *t*-test. * *p* < 0.05, ** *p* < 0.01, *** *p* < 0.001.

We next explored a range of cytokine signaling pathways, as the cytokine production pathway was shown to be downregulated in the RNA-Seq analysis, and cytokines/chemokines are critical in regulating inflammatory response in ECs. Cytokine profiling of 32 cytokines in the supernatants collected from bEND.3 cells after Gm39822 knockdown revealed that over 50% were significantly downregulated (19 of 32 cytokines). This group of downregulated cytokines included IFN-γ, IL-1α, IL-1β, IL-2, IL-3, IL-5, IL-6, IL-17, and MCP-1, which are well described in the literature as key proinflammatory molecules governing several aspects of EC dysfunction, including expression of VCAM-1 and crosstalk between the endothelium and other cellular constituents of the vessel wall (Supplementary Figure S3A–P) [13]. Production of pro- or anti-inflammatory cytokines is regulated by a diverse set of mediators of the NF-κB, P38 MAP kinase, and ERK1/2 signaling pathways [13,14]. Thus, we explored the expression of total and phosphorylated forms of NFKB p65, p50, p38, and ERK1/2 in bEND.3 cells. Western blot analysis showed that knockdown of Gm39822 in bEND.3 cells inhibits the expression of total and phosphorylated protein forms of P65, P38, ERK1/2, and P50 (Supplementary Figure S4A). We further confirmed these findings through immunofluorescence staining, which showed a decrease in p-P65, P65, P50, and P38 upon inhibition of Gm39822 in bEND.3 cells (Supplementary Figure S4B–E). Downstream from this, we quantified the production and secretion of the pro-inflammatory cytokine IL-6. The IL-6 mRNA transcript was downregulated by RTqPCR under both basal conditions and TNF-α stimulation (Supplementary Figure S4F). Similarly, the secretion of IL-6 in the cell supernatant was also decreased upon Gm39822 inhibition (Supplementary Figure S4G). We sought to validate these findings with a broader set of cytokine profiling in mECs and db mECs. The cytokine profiling in cell supernatants collected from mECs showed a similar trend of reduced secretion of cytokines to that observed in bEND.3 cells. In total, 11 out of the 32 detected cytokines were downregulated post-Gm39822 inhibition in mEC supernatants, including the inflammatory cytokines IFN-γ, IL-2, IL-6, and VEGF (Supplementary Figure S5A–K). Further, we examined the impact of Gm39822 knockdown on the expression of total and phosphorylated forms of NFKB p65, p50, p38, and ERK1/2 in mECs and db mECs. A similar trend of inflammatory response to that in bEND.3 cells was observed in mECs after Gm39822 knockdown and overexpression; Gm39822 knockdown repressed the expression of p-NFKB p65, p65, p50, p-p38, p38, p-ERK1/2, and ERK1/2 in mECs (Figure 5A–H). In contrast, the overexpression of Gm39822 in mECs induced the expression of p-NFKB, p65, p65, p50, p-p38, p38, p-ERK1/2, and ERK1/2 (Figure 5I–P). In contrast to the observations in bEND.3 cells and mECs, knockdown of Gm39822 in db mECs tends to induce the expression of inflammatory proteins p-NFKB p65, p65, p50, p-p38, p38, p-ERK1/2, and ERK1/2, particularly after IL-1β treatment (Figure 6A–H). In addition, the secretion of several cytokines increased in db mECs after Gm39822 knockdown, as shown by cytokine profiling; the secretion of 9 of the 35 cytokines profiled increased, and these included Il-12p70, G-CSF, GM CSF, IL-1α, IP-10, LIX, MCP-1, MIP-2, and RANTES post-Gm39822 knockdown in db mECs (Figure 6I–Q).

### 2.5. Endothelial Gm39822 Regulates Migration, Proliferation, Apoptosis, and Angiogenesis in ECs, as Well as the Migration of SMCs

**Figure 7:**
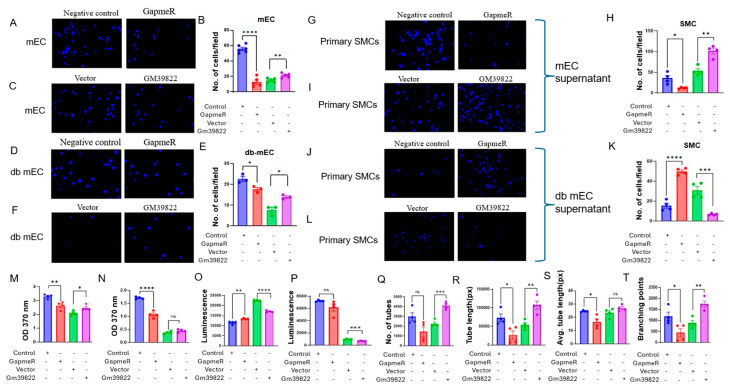
Gm39822 promotes migration, proliferation, and angiogenesis and inhibits apoptosis in mECs. (**A**,**C**) Representative images and (**B**) quantification of migration of non-diabetic endothelial cells (mECs) under a VEGF gradient (50 ng/mL) after (**A**) knockdown or (**C**) overexpression of Gm39822 (*n* = 5/group). (**D** and **F**) Representative images and (**E**) quantification of migration of diabetic endothelial cells (db mECs) under a VEGF gradient (50 ng/mL) after (**D**) knockdown or (**F**) overexpression of Gm39822 (*n* = 5/group). (**G** and **I**) Representative images and (**H**) quantification of the migration of primary smooth muscle cells in response to the cell culture supernatant harvested from non-diabetic endothelial cells (mECs) under a VEGF gradient (50 ng/mL) after (**G**) knockdown or (**I**) overexpression of Gm39822 (*n* = 4/group). (**J**,**L**) Representative images and (**K**) quantification of migration of primary smooth muscle cells in response to the cell culture supernatant harvested from the diabetic endothelial cells (db mECs) under a VEGF gradient (50 ng/mL) after (**J**) knockdown or (**L**) overexpression of Gm39822 (*n* = 4/group). (**M**,**N**) Proliferation as measured with the BrdU assay for (**M**) non-diabetic endothelial cells (mECs) and (**N**) diabetic endothelial cells (db mECs) after knockdown or overexpression of Gm39822; *n* = 4/group. (**O**,**P**) Quantification of caspase 3/7 activity after knockdown or overexpression of Gm39822 in (**O**) non-diabetic endothelial cells (mECs) and (**P**) diabetic endothelial cells (db mECs), *n* = 4/group. (**Q**–**T**) Network tube formation assay and quantification of (**Q**) the number of tubes, (**R**) tube length, (**S**) average tube length, and (**T**) branching points formed in non-diabetic endothelial cells (mECs) after knockdown or overexpression of Gm39822; *n* = 4/group. The data are represented as the mean ± SEM, and statistical significance was determined with an unpaired two-tailed Student *t*-test. ns—non significant, * *p* < 0.05, ** *p* < 0.01, *** *p* < 0.001, **** *p* < 0.0001.

In addition to regulating inflammatory responses, the vascular endothelium modulates homeostatic responses in the vessel wall by impacting cellular proliferation, apoptosis, angiogenesis, and paracrine effects on neighboring vascular smooth muscle cells (VSMCs). First, we quantified the response of Gm39822 on the migration of bEND.3 cells. Knockdown of Gm39822 decreased, whereas overexpression of Gm39822 increased the migration of bEND.3 cells in response to a VEGF gradient in transwell migration assays (Supplementary Figure S6A–C). We further confirmed this finding by performing a scratch assay in the bEND.3 cells following knockdown and overexpression of Gm39822. The findings from the scratch assay were identical to those of the transwell migration assay. The bEND.3 cells were found to migrate slowly following Gm39822 knockdown, as shown by the decreased gap closure after GapmeR treatment, whereas overexpression of Gm39822 increased the migration, as shown by the increased gap closure when compared to their respective controls (Supplementary Figure S6D–H). Furthermore, we tested the role of Gm39822 in regulating proliferation and apoptosis in bEND.3 cells. We quantified BrdU incorporation as a measure of cellular proliferation. Knockdown of Gm39822 decreased, whereas overexpression of Gm39822 increased the proliferation of bEND.3 cells (Supplementary Figure S6I). We also checked the role of Gm39822 in apoptotic cell death by measuring the activity of caspase 3/7 enzymes. Similar to its impact on cellular migration and proliferation, Gm39822 knockdown decreased while overexpression of Gm39822 increased caspase 3/7 activity in bEND.3 cells (Supplementary Figure S6J). We next tested the impact of Gm39822 knockdown and overexpression on mECs and db mECs in transwell migration assays. Similar to the observations in bEND.3 cells, downregulation of Gm39822 decreased, whereas overexpression of Gm39822 increased the migration of mECs and db mECs in response to VEGF (Figure 7A–F).

Apart from endothelial cells, another key event underlying the progression of atherosclerosis is the migration of smooth muscle cells from the media layer into the intima. Endothelial cells contribute to regulating the migration of SMCs into the media, typically in a paracrine manner [15]. Therefore, we next tested whether altering Gm39822 expression in endothelial cells could impact the migratory capacity of primary SMCs. We collected supernatants from mECs and db mECs after knockdown or overexpression of Gm39822. The migration of SMCs was decreased upon Gm39822 knockdown, whereas it was increased upon Gm39822 overexpression in the non-diabetic mECs (Figure 7G–I). In contrast, in the diabetic db mECs, more SMCs migrated upon Gm39822 knockdown, whereas a lower number of SMCs migrated upon Gm39822 overexpression in db mECs (Figure 7J–L).

Following the observations on increased cytokine secretion and migration by Gm39822, we were interested in identifying the impact of Gm39822 on the proliferation of mECs and db mECs. The rationale was that inflammation typically also regulates the proliferation of endothelial cells, which can impact vessel wall function. Interestingly, proliferation, as quantified by BrdU incorporation, was inhibited upon Gm39822 knockdown in both mECs and db mECs, whereas overexpression of Gm39822 showed increased BrdU in mECs but showed no difference in db mECs (Figure 7M,N). To assess effects on apoptosis, we examined the impact of altering Gm39822 expression by measuring caspase 3/7 activity. Gm39822 knockdown increased, whereas overexpression of Gm39822 decreased caspase 3/7 activity in the mECs (Figure 7O). However, a reverse trend was observed in the db mECs, as shown by a non-significant trend of lower caspase 3/7 activity upon Gm39822 knockdown, whereas overexpression of Gm39822 reduced caspase 3/7 activity (Figure 7P). Following the observed changes in increased inflammation, migration, proliferation, and apoptosis, we were curious to examine if these changes led to any visible impact on angiogenic function using a network tube formation assay in mECs. Knockdown of Gm39822 inhibited, whereas the overexpression of Gm39822 promoted tube formation in mECs, as quantified by the increased number of tubes formed (Figure 7Q), tube length (Figure 7R), average tube length (Figure 7S), and number of branching points (Figure 7T). Taken together, these results indicate that Gm39822 increases angiogenic activity in non-diabetic ECs by regulating cellular migration, proliferation, and apoptosis.

### 2.6. Gm39822 Interacts with C1D to Regulate Cellular Processes in Non-Diabetic Endothelial Cells

**Figure 8:**
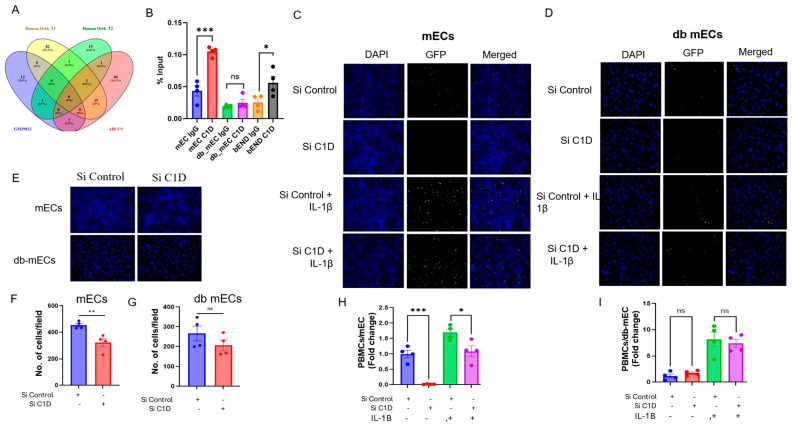
Gm39822 co-localizes and interacts with C1D. (**A**) Venn diagram of interacting partners of Gm39822, transcript 1 and transcript 2 of the lncRNA ENSG00000250994, and the human ABCC9 sequence as identified using the CatRAPID tool. (**B**) Ribonucleoprotein immunoprecipitation (RIP) of Gm39822 using C1D antibody in the lysates obtained from bEND.3 cells, non-diabetic endothelial cells, and diabetic endothelial cells; *n* = 4/group. (**C**,**H**) Representative image (**C**,**H**) quantification of the adhesion of PBMCs in response to siRNA knockdown of C1D in non-diabetic endothelial cells (mECs) under basal conditions and 8 h of IL-1β stimulation (20 ng/mL, *n* = 4/group). (**D**,**I**) Representative image (**D**,**I**) quantification of the adhesion of PBMCs in response to siRNA knockdown of C1D in diabetic endothelial cells (db mECs) under basal conditions and 8 h of IL-1β stimulation (20 ng/mL, *n* = 4/group). (**E**–**G**) Representative images (**E**) and quantification of cellular migration in response to siRNA knockdown of C1D in (**F**) non-diabetic endothelial cells (mECs) and (**G**) diabetic endothelial cells (db mECs), *n* = 4/group). The data are represented as the mean ± SEM, and statistical significance was determined with an unpaired two-tailed Student *t*-test. ns—non significant, * *p* < 0.05, ** *p* < 0.01, *** *p* < 0.001.

Gm39822 is encoded in the mouse genome from the forward strand of chromosome 11 at location Chromosome 11: 51,789,304-51,800,451 (Ensemble, GRCm39). We were interested in knowing if there is an ortholog of Gm39822 in the human genome and the potential mechanism through which Gm39822 exerts its effects in endothelial cells. Gm39822 is flanked by two genes, *JADE2* and *CDKN2AIPNL*. Interestingly, the same genes occur in chromosome 5 of the human genome. Although we did not find Gm39822 annotated as a transcript in the human genome, we did identify a region flanked by *JADE2* and *CDKN2AIPNL,* which encodes a long non-coding RNA, ENSG00000250994. ENSG00000250994 encodes a novel transcript, which could be a potential ortholog of the lncRNA Gm39822, but it did not have sequence similarity. Another human gene, *ABCC9,* encodes a transcript (transcript ID: ENST00000261200.9) that shares ~79% sequence identity in an 86 bp fragment (80292-80374 nucleotides) with the Gm39822 sequence (1278-1364 nucleotides). Therefore, we screened for the potential interacting proteins for Gm39822, ABCC9, and ENSG00000250994 using an online tool, CatRAPID (Figure 8A). Upon careful investigation of proteins that could potentially interact with the transcripts Gm39822, ABCC9, and ENSG00000250994, one potential interactor emerged, the nuclear protein C1D (C1D nuclear receptor corepressor), which is predicted to interact with Gm39822 and ENSG00000250994. C1D has been shown to regulate RNA processing, chromosome condensation, and DNA damage response [16]. Because Gm39822 is a nuclear-enriched lncRNA, we were interested in identifying if there is an interaction between C1D and Gm39822. Therefore, we performed RNA immunoprecipitation assays to validate the C1D–Gm39822 interaction. After RNA pull-down with C1D antibody, we observed that there was a strong interaction between C1D and Gm39822 in bEND.3 cells and mECs, whereas C1D and Gm39822 did not interact in db mECs (Figure 8B). To ascertain the function of C1D under non-diabetic and diabetic conditions, we performed functional assays in mECs and db mECs after siRNA-mediated knockdown of C1D. We first examined the adhesion of PBMCs to ECs. C1D knockdown in mECs inhibited the attachment of PBMCs to mECs (Figure 8C,H). In addition, the transwell migration of mECs was decreased after knockdown of C1D in mECs (Figure 8E,F). Interestingly, knockdown of C1D in db mECs had no impact on either the adhesion of PBMCs to db mECs or the transwell migration of db mECs (Figure 8D,E,G,I). Collectively, these findings suggest that lncRNA Gm39822 may interact with C1D to mediate differential effects in non-diabetic mECs.

## 3. Discussion

Endothelial dysfunction is one of the main factors underlying several cardiovascular disorders, such as atherosclerosis, hypertension, obesity, and diabetes. Despite advances in prevention and current therapeutics, specific strategies to directly reverse endothelial dysfunction remain poorly understood. Advancements in genomics have led to the discovery of a large non-coding fraction in the mammalian genome, creating a surge in research on the potential role of lncRNAs in physiology and disease. This study demonstrates an important role of lncRNA Gm39822 in regulating endothelial functions.

This study demonstrates that Gm39822 is a pro-inflammatory lncRNA under non-diabetic conditions in endothelial cells, whereas in diabetic conditions, it is mildly anti-inflammatory. In support, knockdown of Gm39822 inhibited multiple pathways of inflammatory response, decreased cytokine secretion, and inhibited leukocyte attachment, migration, and proliferation of non-diabetic endothelial cells. In contrast, in diabetic endothelial cells, Gm39822 did not have an impact on the monocyte attachment and proliferation, although it increased the migration of ECs similarly to under nondiabetic conditions. Furthermore, we identified C1D as an important interacting partner of Gm39822 that could be a causal agent of the changes brought by Gm39822 under non-diabetic conditions. The finding that C1D does not interact with Gm39822 in diabetic endothelial cells may be one of the reasons underlying the relatively small impact of Gm39822 on its cellular functions.

We have previously discovered several lncRNAs that mediate cell-specific roles in vascular inflammation, such as the macrophage-enriched lncRNAs MERRICAL and MAARS [9,17], the vascular-smooth-muscle-cell-enriched lncRNA CARMN [18], and the endothelial-enriched lncRNAs SNHG12 and VINAS [7,8]. Similarly, other studies have highlighted the expression and function of lncRNAs in a cell-type-specific manner [19,20,21]. These examples underscore the importance of lncRNAs in cardiovascular pathology and suggest that identifying novel lncRNAs with cell-specific regulatory functions could provide valuable insights into disease mechanisms.

In this study, we show differential regulation of endothelial dysfunction in diabetic and non-diabetic endothelial cells by Gm39822. Diabetic and non-diabetic cells can have significantly different gene expression signatures, resulting in profoundly altered key molecular mechanisms underlying the cellular dysfunctions, leading to atherosclerosis and other cardiovascular diseases. Studies using RNA-seq and microarray profiling techniques have identified significant alterations in genes related to insulin signaling, inflammation, oxidative stress, proliferation, and angiogenesis in diabetic endothelial cells or tissues from diabetic patients as compared to patients without diabetes [22,23]. For example, differential gene expression or epigenetic modifications in disease-associated transcripts such as *INSR* (insulin receptor) and *PPARG* (peroxisome proliferator-activated receptor gamma) can regulate glucose uptake, and metabolic dysregulation may be more pronounced in diabetic ECs than non-diabetic ECs [24,25]. Accumulating studies highlight the importance of the non-coding genome, including lncRNAs, as emerging mediators of cell-specific regulatory programs [26]. While the majority remain poorly characterized, lncRNAs play a crucial role in cellular metabolism by modulating key regulatory mechanisms, including chromatin remodeling, transcriptional control, and post-transcriptional modifications [15,21,27]. Important aspects of lncRNA biology that may influence their mechanism and function in cells include the relative quantity of lncRNA expression, subcellular localization, and the extent of cell-type-specific expression.

In this work, we identified Gm39822 as a nuclear-enriched lncRNA that is particularly abundant in non-diabetic endothelial cells. While the extent of expression can be a critical contributor to the function of Gm39822, we also found that Gm39822 interacts with a nuclear receptor, C1D, specifically in non-diabetic cells and not in diabetic cells. This potentially is reflected in a range of cellular functions that are contrastingly regulated by Gm39822 in non-diabetic compared to diabetic endothelial cells. We investigated some of the core biological functions involving endothelial cells, namely, the expression of cell-surface adhesion proteins, immune cell recruitment, inflammation, and signaling pathways. The recruitment of immune cells to the endothelium is a pivotal event in the progression of cardiovascular disorders, involving various adhesion molecules, with VCAM-1 serving as a key mediator of this process. VCAM-1 is an inducible adhesion molecule responding to various stimuli, including pro-inflammatory cytokines, reactive oxygen species, shear stress, and oxidized low-density lipoprotein [28,29,30]. Our study shows an important role of Gm39822 in regulating cellular inflammation and its downstream effects. In non-diabetic ECs, Gm39822 acts as a pro-inflammatory lncRNA, inducing the expression of several inflammatory pathways mediated by NF-κB (p65, p50) and the MAP kinase ERK1/2 and P38 pathways in non-diabetic mECs and bEND.3 cells (Figure 5 and Supplementary Figure S4). NF-κB is a central transcription factor that rapidly induces pro-inflammatory cytokine expression upon activation in a positive feedback loop, whereas members of the MAPK family (ERK1/2 and p38 MAPK) also modulate cytokine production—both of these pathways were induced by Gm39822 in non-diabetic cells. Expectedly, the knockdown of Gm39822 inhibited several of the proinflammatory cytokines in non-diabetic mECs and bEND.3 cells (Supplementary Figures S3 and S5). Notable cytokines that were consistently downregulated in both bEND.3 and non-diabetic endothelial cells included Eotaxin, G-CSF, IFN-γ, IL-2, IL-6, and IP-10. These cytokines are known to induce VCAM-1 expression and the recruitment of immune cells in various diseases. For instance, eotaxin recruits eosinophils through VCAM-1, leading to tissue eosinophilia [31]. Similarly, G-CSF [32], IFN-γ [33], and IL-6 [34] increase the expression of VCAM-1 and promote the adhesion of immunoregulatory and hematopoietic cells. In line with decreased cytokine production, Gm39822 knockdown decreased VCAM-1 expression in both bEND.3 cells and non-diabetic ECs. Consistently, there was reduced attachment of PBMCs to these non-diabetic ECs. In contrast, Gm39822 exhibited an anti-inflammatory role in diabetic endothelial cells (Figure 2 and Figure 3). The knockdown of Gm39822 increased the expression of NF-KB p65, p50, ERK1/2, and P38, and consequently, the cytokine secretion increased in diabetic endothelial cells (Figure 6). However, this did not reflect in any significant increase in VCAM-1 expression or PBMCs’ adhesion to db mECs. One of the reasons could be that many of the screened cytokines were also upregulated after Gm39822 knockdown in db mECs. Because there was no interaction between Gm39822 and C1D in diabetic ECs, there could possibly be other regulatory mechanisms governing inflammation in these cells, which are subject to further investigation.

C1D, the interacting partner of Gm39822 in non-diabetic endothelial cells, is a DNA-binding corepressor localized in the nucleus. C1D is known to regulate multiple cellular processes, including DNA repair, RNA processing, and oxidative stress responses [35,36]. We found that C1D also regulated leukocyte recruitment, as well as the migration of non-diabetic endothelial cells. We identified C1D as a potential interacting partner of Gm39822 following a set of bioinformatics analyses intended to identify a potential ortholog of Gm39822 in the human genome. Gm39822 shows sequence similarity of approximately 79% to an 86 bp sequence stretch of ABCC9 in the human genome. ABCC9 encodes a critical component of ATP-sensitive potassium channels of the ATP-binding cassette (ABC) transporter superfamily, enriched in cardiac, skeletal, vascular, and non-vascular smooth muscle. Mutations in ABCC9 are associated with dilated cardiomyopathy type 10 and familial atrial fibrillation (type 12). C1D has been identified as a DNA double-strand break repair protein that interacts with exosomal subunits PM/Scl-100, and it is involved in the regulation of pre-RNA processing [35], oxidative stress [36], and UV-induced apoptosis [37]. This implicates the potential role of C1D in cardiovascular disorders; however, we did not find any relevant studies in the literature depicting C1D in endothelial dysfunction or atherosclerosis. Future studies will be required to further elucidate the role of C1D in cardiovascular disease models. In line with the findings of Gm39822 knockdown on EC migration, C1D knockdown also inhibited the migration of non-diabetic mECs. Interestingly, C1D did not affect the migration of diabetic mECs (Figure 8E,G). Furthermore, using supernatants from ECs, altered endothelial Gm39822 levels impacted SMCs in a paracrine manner by altering their ability to migrate. Whereas the trend of the migration of SMCs following C1D knockdown was identical to that of Gm39822 knockdown in non-diabetic mECs, it differed from that of diabetic mECs post-Gm39822 knockdown or overexpression. This indicates that factors other than C1D could be responsible for regulating the migration of SMCs in response to Gm39822 levels in diabetic mECs. Furthermore, siRNA-mediated knockdown of C1D prohibited the attachment of PBMCs to non-diabetic mECs, whereas C1D knockdown did not have any impact on the attachment of PBMCs to diabetic mECs (Figure 8C,D,H,I). This again corresponded well with the observations of reduced attachment of PBMCs to non-diabetic mECs and no impact of Gm39822 on the attachment of PBMCs to diabetic mECs. These findings indicate a likely spatial and functional correlation between Gm39822 and C1D that may be context-dependent.

## 4. Materials and Methods

### 4.1. RNA Isolation and qRT-PCR

Trizol reagent (15596-026, Invitrogen, Waltham, MA, USA) and miRNeasy Tissue/Cells Advanced Kits (217684, Qiagen, Germantown, MD, USA) were used for the isolation of total RNA from the cells, following the manufacturer’s instructions. NanoDrop 2000 (ThermoFisher, Waltham, MA, USA) was used for quantifying RNA. High-Capacity cDNA Reverse Transcription Kit (4368814, Thermofisher, Waltham, MA, USA) was used for cDNA synthesis. mRNA expression levels were normalized to β-actin. Quantitative real-time PCR was performed using Quantstudio 6 Pro (Thermofisher, Waltham, MA, USA) with GoTaq qPCR (A6001, Promega, Madison, WI, USA) while following the manufacturer’s instructions. The list of primers is presented in Table S1.

### 4.2. Cellular Fractionation

Cytoplasmic and nuclear RNA was separated using a nuclear extract kit (Active Motif, 40010) and cleaned up using the RNeasy kit (74204, Qiagen, Germantown, MD, USA) while following the manufacturer’s instructions. RNA in the nuclear and cytoplasmic fractions was quantified using qRT-PCR, as described previously [9].

### 4.3. Protein Coding Potential

The in silico CPAT online algorithm was used for the prediction of the coding potential of the lncRNAs.

### 4.4. RNA In Situ Hybridization (RNA-ISH)

Gm39822 was detected using a customized probe designed to detect Gm39822 (1175841-C1, Advanced Cell Diagnostics, Newark, CA, USA). Cells were fixed for 2 h in 4% paraformaldehyde and further prepared as described by the manufacturer. RNA-ISH was performed using RNAscope 2.5 HD Reagent Kit-Red (322372, Advanced Cell Diagnostics, Newark, CA, USA) while following the manufacturer’s protocol.

### 4.5. Molecular Cloning for Gm39822 Overexpression

The entire sequence of the Gm39822 transcript (Gene ID: 105244162) was synthesized (Genewiz, Waltham, MA, USA) and cloned into the pcDNA.3.1 plasmid using EcoRI and XhoI restriction sites. After validation of sequencing, the plasmids were used for transient transfection assays in the indicated cell types, as detailed below.

### 4.6. Cell Culture and Transfection

bEnd.3 cells (CRL-2299, ATCC, Manassas, VA, USA) were cultured in Dulbecco’s Modified Eagle Medium/F12(1:1) (DMEM; Gibco, 11320-033, Waltham, MA, USA) supplemented with 10% fetal bovine serum (FBS) and 1% penicillin–streptomycin (P/S). Non-diabetic (mECs, C57-6220, Cell Biologics, Chicago, IL, USA) and diabetic (db mECs, MD-6220, Cell Biologics, Chicago, IL, USA) mouse skeletal muscle endothelial cells were cultured in endothelial cell medium (M1168, Cell Biologics, Chicago, IL, USA), supplemented as directed by the supplier. MOVAS cells were cultured in DMEM (10566016, Gibco, Grand Island, NY, USA) supplemented with 10% FBS and 1% P/S. Primary mouse aortic SMCs were cultured in smooth muscle growth media (SmBm basal media (CC-3181, Lonza, Walkersville, MD, USA) supplemented with SmGm supplements (CC-4149, Lonza, Walkersville, MD, USA), 10% final FBS, and 1%P/S, and they were used for between 3 and 6 passages. Transfections were performed using Lipofectamine 2000 (11668019, Invitrogen, Rockford, IL, USA) as described in the manufacturer’s protocol. For knockdown studies of Gm39822, custom antisense LNA GapmeRs targeted to Gm39822 (Cat. No. LG00798344-DDA, Qiagen, Valencia, CA, USA;100 nM) or a negative control antisense LNA GapmeR (Cat. No. LG00000002-EFA, Qiagen, Valencia, CA, USA; 100 nM) were used. For knockdown studies of C1D, siRNA targeting C1D (L-051454-01-0005, Horizon, Boulder, CO, USA) or a negative control siRNA was used (D-001810-10-05, Horizon, Boulder, CO, USA). For overexpression studies, a control vector (pcDNA3.1, 1000 ng/mL) or a vector containing Gm39822 (1000 ng/mL) was used for transfections.

### 4.7. Cell Adhesion Assay

Peripheral blood mononuclear cells (PBMCs) were harvested from wild-type C57BL/6 mice using a lymphocyte separation medium (50494, MP Biomedicals, Solon, OH, USA) while following the manufacturer’s protocol. Harvested PBMCs were resuspended in growth medium containing 5 μM Calcein AM (C3100MP; Invitrogen, Carlsbad, CA, USA) to achieve the final density of 5 × 10^6^ cells/mL and were kept for 30 min in an incubator at 5% CO_2_ and 37 °C. Cells were washed with growth medium twice and resuspended in Calcein-loaded growth medium at 5 × 10^6^ cells/mL. TNF-α (20 ng/mL) was treated for 8 h. Following this, 500 μL of Calcein-AM-loaded PBMCs were added per well to each cell type and incubated for 1 h. Cells were carefully washed four times with warm growth media after incubation to remove the nonadherent PBMCs. Images of cells stained with Calcein AM and DAPI were captured using a Keyence fluorescence microscope. Cells were counted using the Image-J software, 1.54p.

### 4.8. Western Blotting and Immunocytochemistry

Cells were lysed and proteins were isolated using RIPA lysis buffer (BP-115, Boston BioProducts, Ashland, MA, USA) containing a 1× protease and phosphatase inhibitor cocktail (5872, CST, Danvers, MA, USA). The total protein concentration was estimated using the Pierce BCA assay kit (23228, Thermo Fisher Scientific, Waltham, MA, USA). Then, 20 μg of total protein was separated over a 4–20% Mini-PROTEAN TGX Gel (456-1096, Bio-Rad, Hercules, CA, USA). A Turbo Blot system (Bio-Rad) and Trans-Blot Turbo Transfer Kit (170-4272, Bio-Rad, Hercules, CA, USA) were used to transfer the SDS-resolved protein to a PVDF membrane. Membranes were blocked using 5% nonfat milk in TBST or 5% BSA in TBST for 1 h at room temperature, as required by the specific antibody. Following blocking, the membranes were incubated overnight at 4 °C or for 1.5 h at room temperature with the antibodies VCAM-1 (ab134042, Abcam, Cambridge, UK; 1:1000), NFKB-p65 (SC 8008, Santa Cruz Biotechnology, Dallas, TX, USA; 1:500), p-NFKB-p65 (3033S, CST, Danvers, MA, USA; 1:1000), P50 (SC 8414, 1:500, Santa Cruz Biotechnology, Dallas, TX, USA), p-P38MAPK (4511S, CST, Danvers, MA, USA; 1:1000), P38MAPK (9212S, CST, Danvers, MA, USA; 1:1000), ERK1/2 (9102, CST, Danvers, MA, USA; 1:1000), p-ERK1/2 (4370, CST, Danvers, MA, USA; 1:1000), and b-actin (15739, Invitrogen, Grand Island, NY, USA; 1:5000). Quantification of images was accomplished using Image-J software, 1.54p.

### 4.9. Scratch Assay and Transwell Cell Migration Assay

For the scratch assay, bEND.3 cells were seeded at a density of 100,000 cells/well in a 6-well plate and transfected as described previously [38]. Then, 24 h after transfection, cells were harvested and reseeded at a density of 20,000 cells per quadrant of a 4-chambered culture insert (80469, ibidi, Martinsried, Germany). Transfections were continued for another 24 h. The inserts were lifted after 24h, and cells were washed once carefully with fresh culture media. Images were captured every hour up to 48 h or until the closure of the scratch using CytoSMART Omni (Cytosmart, Axion BioSystems, Amsterdam, The Netherlands). Similarly, for the transwell migration assay, endothelial cells were seeded and transfected, as described previously [38]. After 24 h of transfections, the cells were trypsinized, and 20,000 cells were seeded over an 8 μm pore size insert. Cells were allowed to migrate in response to VEGF (20 ng/mL) across the membrane for 16 h. Thereafter, non-migrated cells were removed from the top of the insert using a cotton swab, and migrated cells were fixed and stained with DAPI. Images were captured using a Keyence fluorescence microscope, and cells were counted using the Image-J software, 1.54p. For the transwell migration of smooth muscle cells (MOVAS and primary smooth muscle cells), endothelial cells were seeded and transfected, as described previously. After 48 h of transfection, the supernatant was collected, and debris was removed by centrifugation at 3000× *g* for 5 min. MOVAS and primary smooth muscle cells were cultured separately and were trypsinized after 24 h of seeding. Then, 20,000 cells were seeded over the transwell inserts and allowed to migrate in response to the supernatant collected from endothelial cells for 16 h. Subsequently, downstream processing of cells, as well as data acquisition and analysis, was performed as described above for endothelial cells.

### 4.10. Bromodeoxyuridine (BrdU) Incorporation Assay

Endothelial cells were seeded and transfected as described previously for the scratch and transwell migration assays [38]. Cells were harvested and reseeded at a density of 10,000 cells/well on a 96-well plate after 24 h of transfection. BrdU was added to cells after 18 h and continued for another 6 h. Cellular proliferation was measured using a Cell Proliferation ELISA BrdU Colorimetric Kit while following the manufacturer’s protocol (11647229001, Roche, Basel, Switzerland).

### 4.11. Ribonucleoprotein Immunoprecipitation (RIP) Assay

A native RIP assay was performed to confirm the interaction of Gm39822 and C1D in endothelial cells, as described previously [9]. Briefly, 1 × 10^7^ endothelial cells were collected and lysed in polysome lysis buffer. Then, 10 μL of the lysate was used as input. Protein A/G Magnetic Beads (88803, ThermoFisher scientific 88803, Rockford, IL, USA) were coated with 5 μg of either anti-C1D or anti-IgG (2727, CST, Danvers, MA, USA) antibodies for 1 h at room temperature. The antibody-coated beads were washed and incubated with 100 μL of cell lysate overnight at 4 °C. Subsequently, the beads were collected and reconstituted in 150 μL of Proteinase K buffer and incubated for 30 min at 55 °C. After protein lysis, cellular RNA was isolated using the phenol:chloroform:isoamyl alcohol (125:24:1) method. Subsequently, cDNA synthesis and qRT-PCR were performed using primers specific for Gm39822, as described previously [9].

### 4.12. Caspase 3/7 Activity Assay

Endothelial cells were seeded and transfected, as described previously [38]. Cells were harvested and reseeded at a density of 10,000 cells/well on a 96-well plate after 24 h of transfection. After 48 h of transfection, caspase 3/7 activity was measured using Caspase-Glo^®^ 3/7 Assay System according to the manufacturer’s instructions (G8090, Promega, Madison, WI, USA).

### 4.13. Cytokine Profiling in Cell Culture Supernatants

Cytokine profiling was performed on supernatants collected from transfected bEND.3 cells, mECs, and db mECs, seeded at 1 × 10^6^ cells per well on a 6-well plate. Following transfections, supernatants were collected, and debris was removed by centrifugation at 3000× *g* for 15 min at 4 °C. Supernatants were stored at −80 °C until further use. Supernatants were subjected to the Mouse Cytokine/Chemokine 31-Plex Discovery Assay Array (MD32, Eve Technologies, Calgary, AB, Canada).

### 4.14. RNA-Seq Analysis

Bulk RNA sequencing was performed in bEND.3 cells after ribodepletion and library construction by using standard library construction and Illumina HiSeq2500 V4 2 × 150 PE (Azenta, West Conshohocken, PA, USA). Samples were processed by using a pipeline published in the bcbio-nextgen project (https://github.com/bcbio/bcbio-nextgen, assessed date 29 September 2023). Raw reads were filtered and examined for quality control by running FastQC (http://www.bioinformatics.babraham.ac.uk/projects/fastqc/, assessed date 29 September 2023), and filtered reads were used to generate the library and for further analysis. Trimmed reads were aligned to UCSC build mm10 of the mouse genome and augmented with transcript information from Ensembl using STAR. All samples had >90% of mapped fragments over total fragments. The RNA-Seq dataset comprised 4 samples each for the GapmeR and negative control groups. A data quality assessment was performed by clustering samples based on gene expression profiles and then visualizing the principal components, PC1 and PC2, on a 2D plane. The results indicated that the two groups were well separated from each other on the x-axis (97% variance), without the presence of any outlier samples. Before the differential expression analysis, pre-filtering of the dataset was also performed to keep only transcripts with a count of at least 5 for a minimal number of samples. Total gene hit counts and CPM values were calculated for each transcript, and differential expression analysis between specified groups was performed using DESeq2 v1.42.1 [39]. A Wald test was used for hypothesis testing during differential expression, and the differentially expressed transcripts (DETs) were selected based on the *p*-value (>0.05) and a fold change of 1.5, where >1.5 and <−1.5 represent up- and downregulated transcripts, respectively, in GapmeR samples.

### 4.15. Pathway Enrichment Analysis

Pathway enrichment analysis of DETs was performed using the QIAGEN-Ingenuity Pathway Analysis (IPA) software (www.ingenuity.com). Fisher’s Exact Test was used to calculate the statistical significance of the overlap of differentially expressed transcripts with canonical pathways, and the pathways were selected based on their statistical significance (*p*-value < 0.05) and enrichment (Z) scores. All analyses were performed in the R v4.3.3 (www.r-project.org) statistical environment, and the plots were generated with the ggplot2 v3.5.1 (ggplot2.tidyverse.org) library.

### 4.16. Study Approval

All animal protocols were conducted in accordance with the NIH Guide for the Care and Use of Laboratory Animals (National Academies Press, 2011) and were approved by the Institutional Animal Care and Use Committee at Brigham and Women’s Hospital and Harvard Medical School. We used C57BL/6 male mice (Charles River) for the experiments.

### 4.17. Statistical Analysis

We used an unpaired non-parametric Student *t*-test to determine statistical significance between two groups using the GraphPad 7.0 software package (GraphPad Software, Inc., San Diego, CA, USA). The data are expressed as the mean ± SEM. We considered a *p*-value of <0.05 as significant for all tests.

## 5. Conclusions

In summary, this study demonstrated a novel role of the lncRNA Gm39822 in regulating endothelial function and its contrasting impact in diabetic and non-diabetic ECs. Our findings revealed that Gm39822 acts as a pro-inflammatory lncRNA in non-diabetic endothelial cells, promoting cytokine secretion, leukocyte adhesion, and endothelial migration while exhibiting a contrasting anti-inflammatory role in diabetic endothelial cells. The interaction of Gm39822 with the nuclear receptor C1D appears to be a key regulatory mechanism in non-diabetic conditions, influencing multiple inflammatory pathways, including NF-κB and MAPK signaling. The absence of this interaction in diabetic endothelial cells may underlie the altered inflammatory response and functional outcomes observed in this setting. Furthermore, our results highlight the broader impact of Gm39822 on EC-SMC crosstalk, suggesting that it may have a potential role in vascular remodeling and atherosclerosis progression. The identification of C1D as a functional partner of Gm39822 provides new insights into endothelial dysfunction, emphasizing the importance of lncRNA-mediated transcriptional regulation in vascular biology. These findings open new avenues for targeted therapeutic interventions, particularly in the context of endothelial dysfunction associated with cardiovascular diseases under non-diabetic conditions. Future studies exploring the translational potential of Gm39822 modulation could provide valuable strategies for mitigating vascular inflammation and its pathological consequences.

## Data Availability

All relevant data are available from the authors. The RNA-seq data are accessible in the following GEO dataset: GSE305264.

## Supplementary Materials

The following supporting information can be downloaded at: https://www.mdpi.com/article/10.3390/ijms26178147/s1.
