# Supplementary material for: Endothelial-Enriched lncRNA Gm39822 Modulates Inflammation and Dysfunction in Non-Diabetic Endothelial Cells
Source: Int J Mol Sci. 2025 Aug 22;26(17):8147. doi: 10.3390/ijms26178147 (PMC12427768; doi:10.3390/ijms26178147)
Supplement: Supplementary file 1 [file ijms-26-08147-s001.zip › 1-ijms-3810727-supplement-8.22/GM39822 SUPPL_Figures_Final.pdf]

Supplementary Figure S1

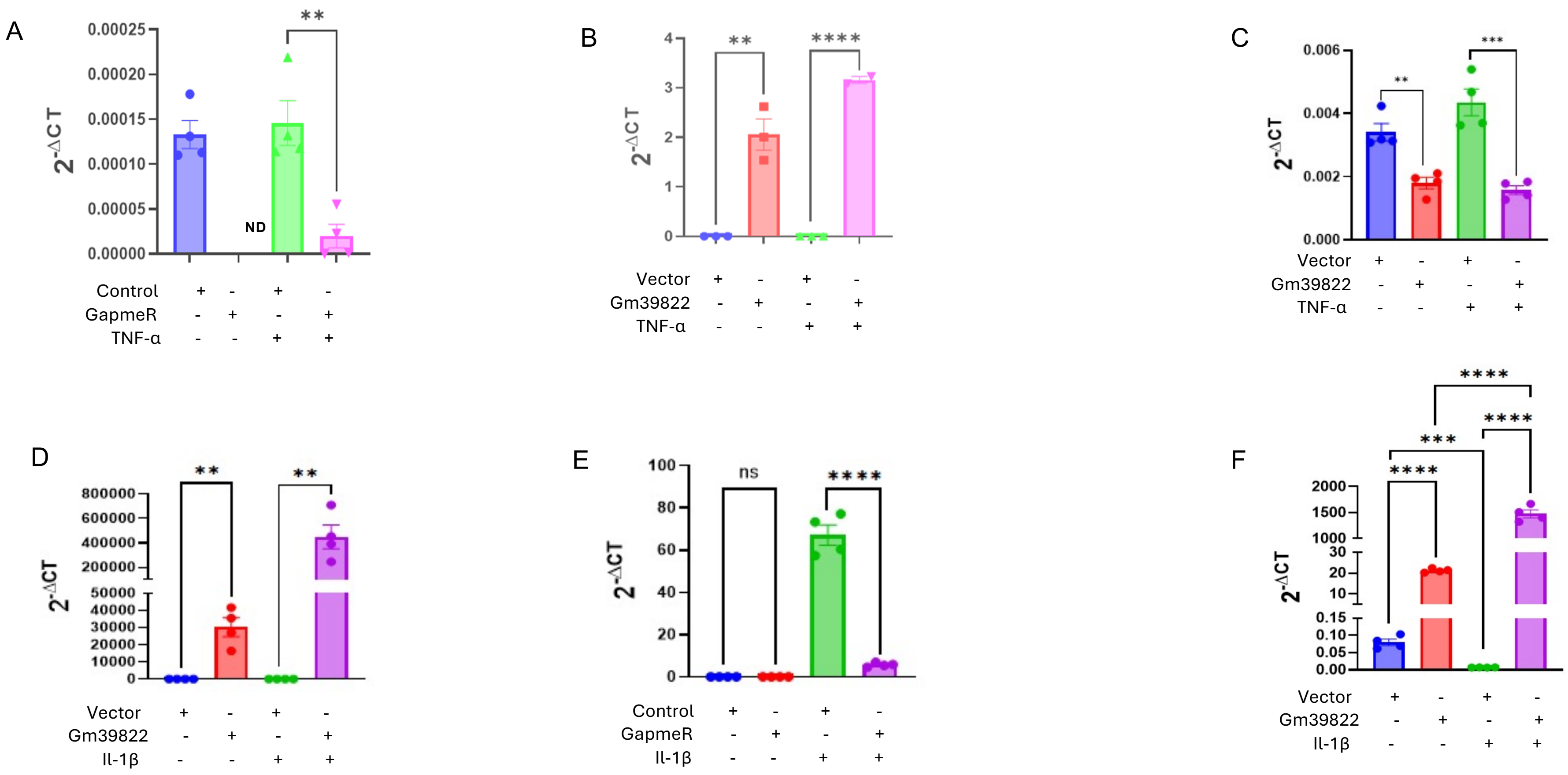

Supplementary Figure S1. Gm39822 knockdown and overexpression in bEND.3, mECs, and db mECs.

qPCR analysis of Gm39822 expression after (A) GapmeR treatment or (B) overexpression in bEND.3 cells. Gm39822 knockdown and overexpression in mECs (C-D) and db mECs (E-F), n=4/group. The data is represented as mean ± SEM and statistical significance was determined by unpaired two-tailed Student's t test. *$p < 0.05$, **$p < 0.01$, ***$p < 0.001$, ****$p < 0.0001$.

Supplementary Figure S2. Gm39822 regulates VCAM1 expression and adhesion of PBMCs to bEND.3 cells

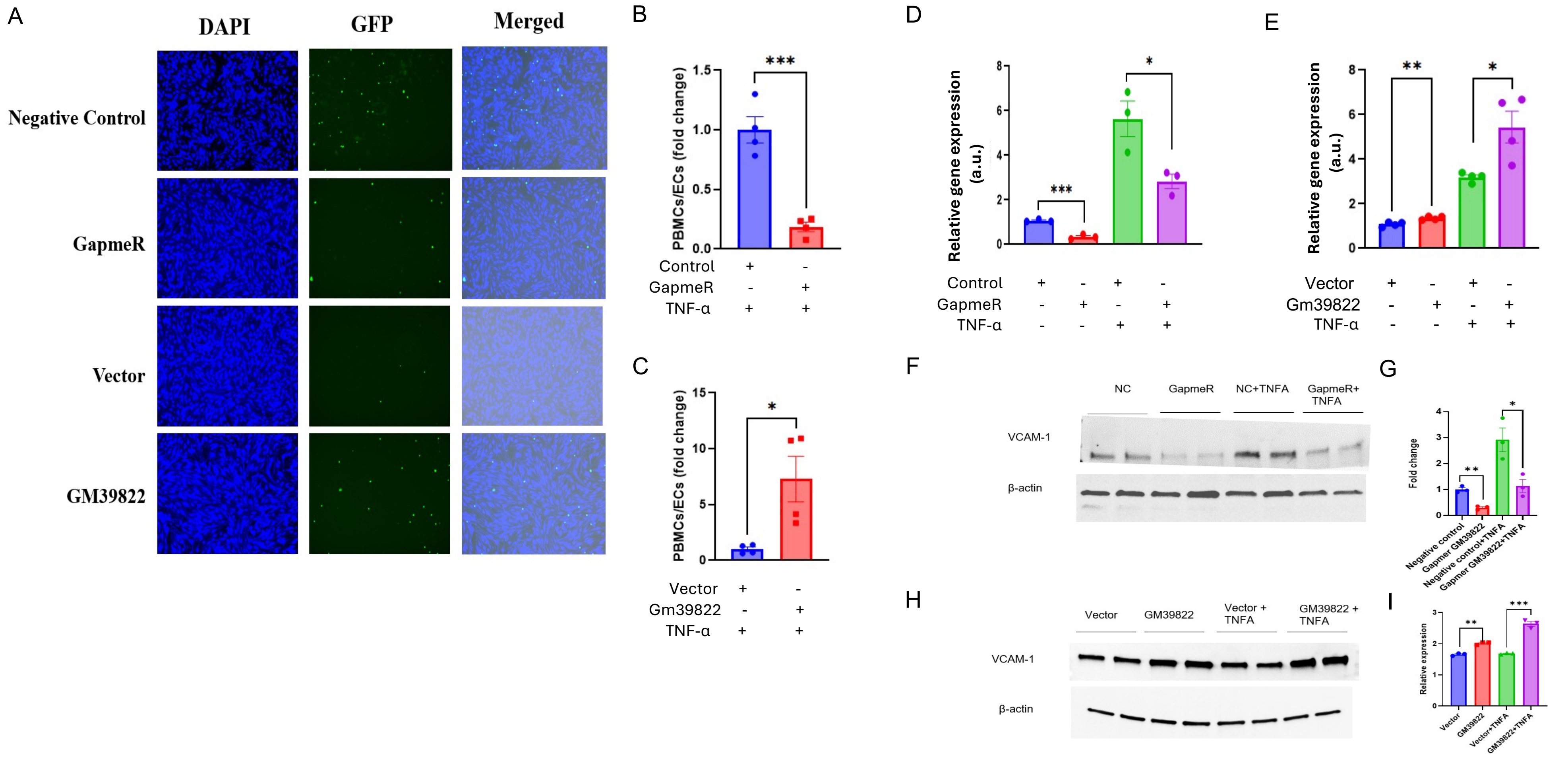

Supplementary Figure S2. Gm39822 regulates VCAM1 expression and adhesion of PBMCs to bEND.3 cells

(A) Representative images and (B and C) quantification of PBMCs adhesion to the bEND.3 cells after knockdown and overexpression of Gm39822, n=4/group.

(D-E) Quantification of VCAM–1 mRNA in bEND.3 cells under basal and TNF-α treatment (10 ng/mL for 2 hours) after (D) knockdown and (E) overexpression of Gm39822, n=4/group.

(F) Representative images and (G) quantification of VCAM–1 expression at protein levels by Western blot analysis in bEND.3 cells under basal and TNF-α treatment (10 ng/mL for 8 hours) after knockdown of Gm39822, n=3/group.

(H) Representative images and (I) quantification of VCAM–1 expression at protein levels by Western blot analysis in bEND.3 cells under basal and TNF-α treatment (10 ng/mL for 8 hours) after overexpression of Gm39822, n=3/group. The data is represented as mean ± SEM and statistical significance was determined by unpaired two-tailed Student's t test. *$p < 0.05$, **$p < 0.01$, ***$p < 0.001$.

Supplementary Figure S3

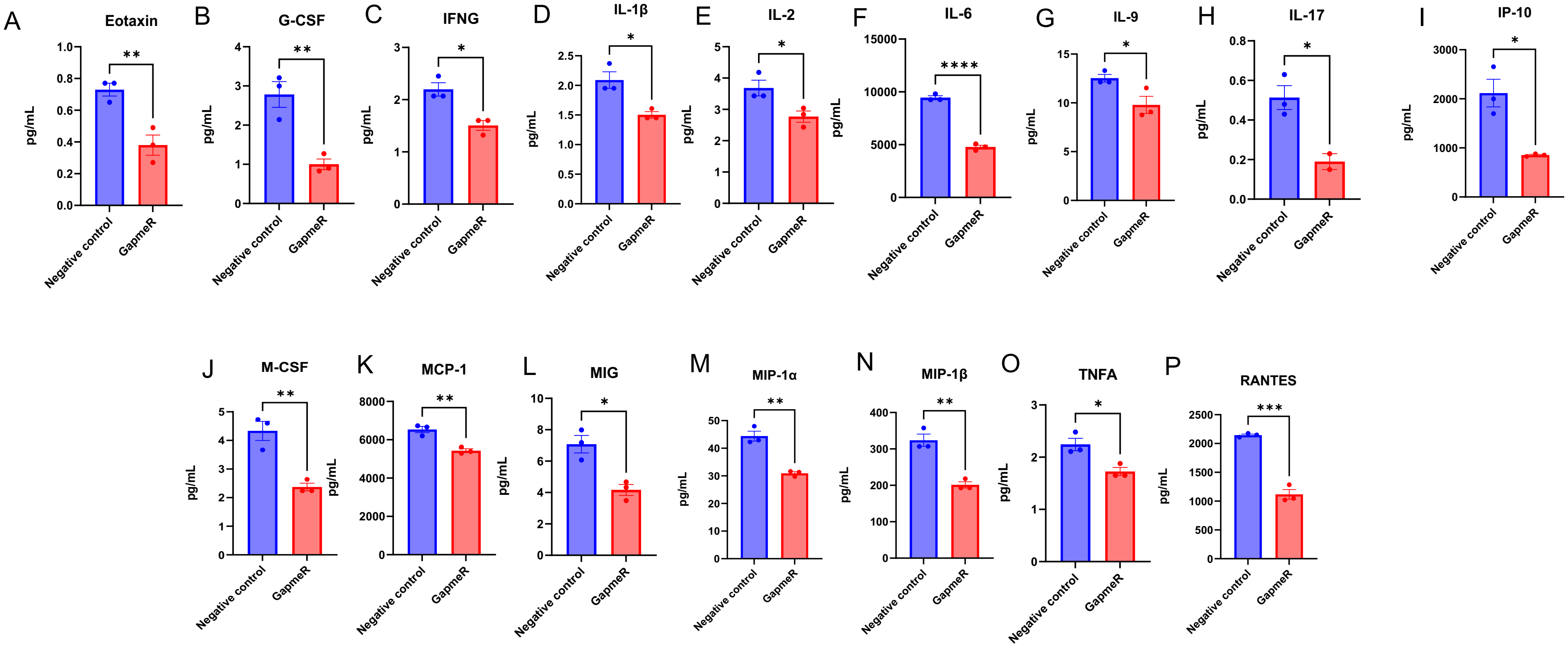

Supplementary Figure S3. Gm39822 knockdown inhibits cytokine secretion in bEND.3 cells

Cytokine profiling was performed of supernatants collected after Gm39822 knockdown in the bEND.3 cells and the following cytokines were significantly reduced:: (A) Eotaxin, (B) G-CSF, (C) IFNG, (D) IL-1b, (E) IL-2, (F) IL-5, (G) IL-9, (H) IL-17, (I) IP-10, (J) M-CSF, (K) MCP1, (L) MIG, (M) MIP-1a, (N) MIP-1b, (O) TNFA, and (P) RANTES, n=3/group. The data is represented as mean ± SEM and statistical significance was determined by unpaired two-tailed Student's t test. *p < 0.05, **p < 0.01, ***p < 0.001.

Supplementary Figure S4

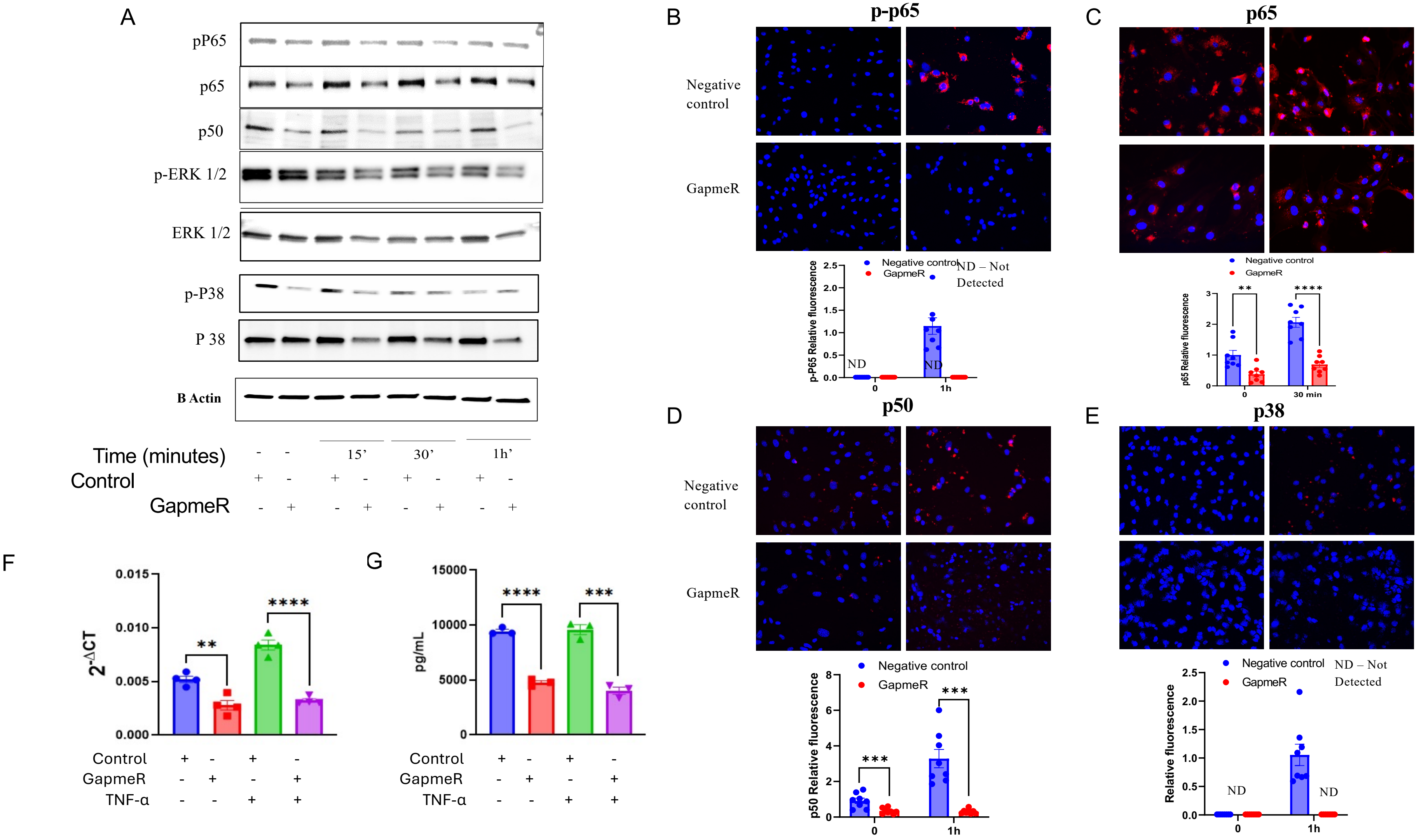

Supplementary Figure S4. Inhibition of Gm39822 downregulates inflammation in bEND.3 cells

(A) Western blots of p-p65, p65, p50, p-ERK1/2, ERK1/2, p-p38, and p38 expression at the indicated time points under basal or TNF-α stimulation (20 ng/mL, n=1/group) after knockdown of Gm39822 in the bEND.3 cells.

(B-E) Representative images and quantification of (B) p-p65, (C) p65, (D) p50, and (E) p38 by Immunofluorescence imaging, n=8/group.

(F-G) Quantification of IL-6 by (F) qPCR and (G) cytokine profiling in the bEND.3 cells. The data is represented as mean ± SEM and statistical significance was determined by unpaired two-tailed Student's t test. *p < 0.05, **p < 0.01, ***p < 0.001, ****p < 0.0001..

Supplementary Figure S5

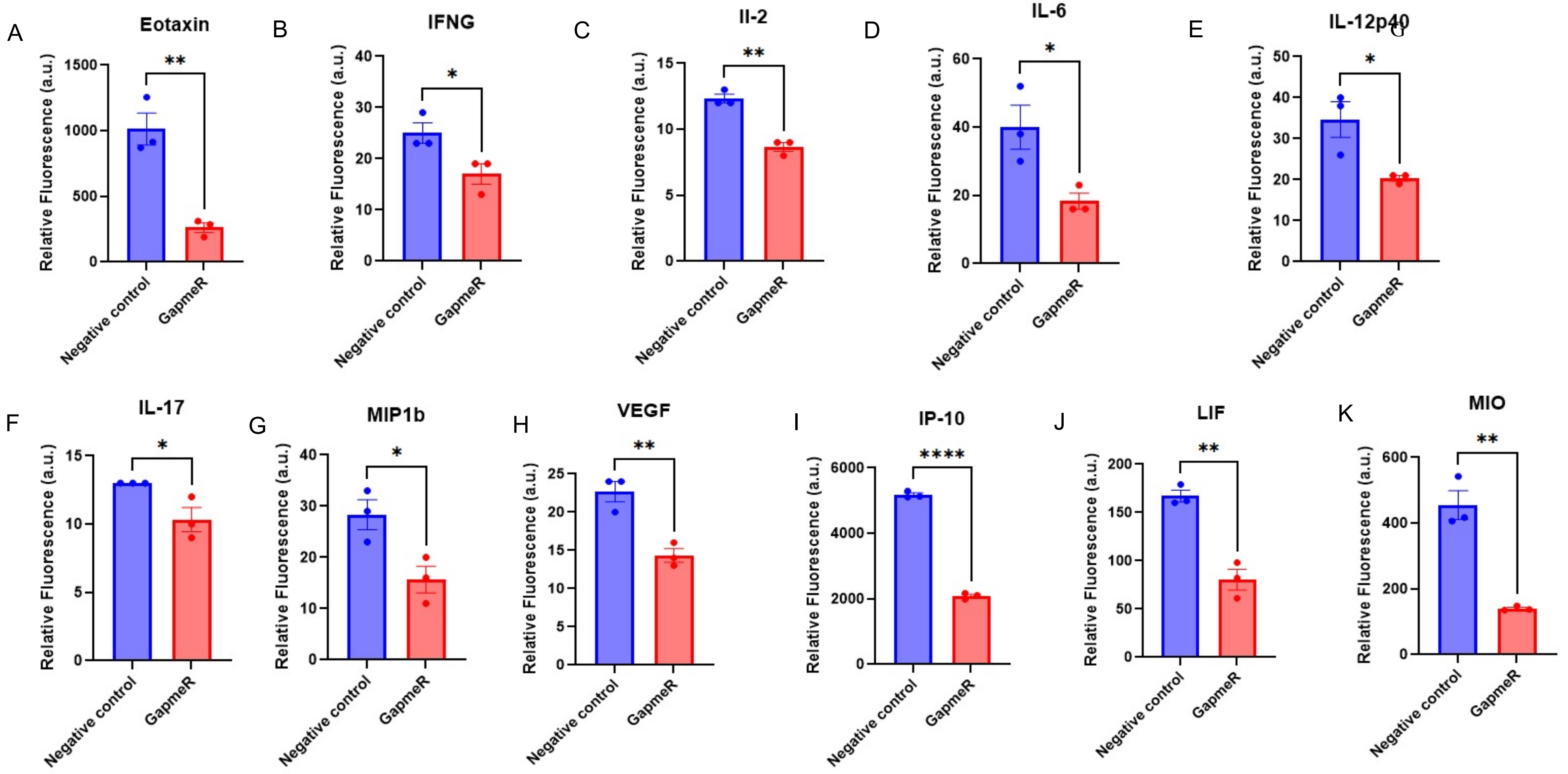

Supplementary Figure S5. Gm39822 knockdown inhibits cytokine secretion in mECs

Cytokine profiling was performed of supernatants collected after Gm39822 knockdown in the non-diabetic endothelial cells and the following cytokines were significantly reduced: (A) Eotaxin, (B) IFNG, (C) IL-2, (D) IL-6, (E) IL-12p40 (F) IL-17, (G) MIP-1b, (H) VEGF, (I) IP-10, (J) LIF, and (K) MIO, n=3/group. The data is represented as mean ± SEM and statistical significance was determined by unpaired two-tailed Student's t test. *p < 0.05, **p < 0.01, ***p < 0.001, ****p < 0.0001..

Supplementary Figure S6

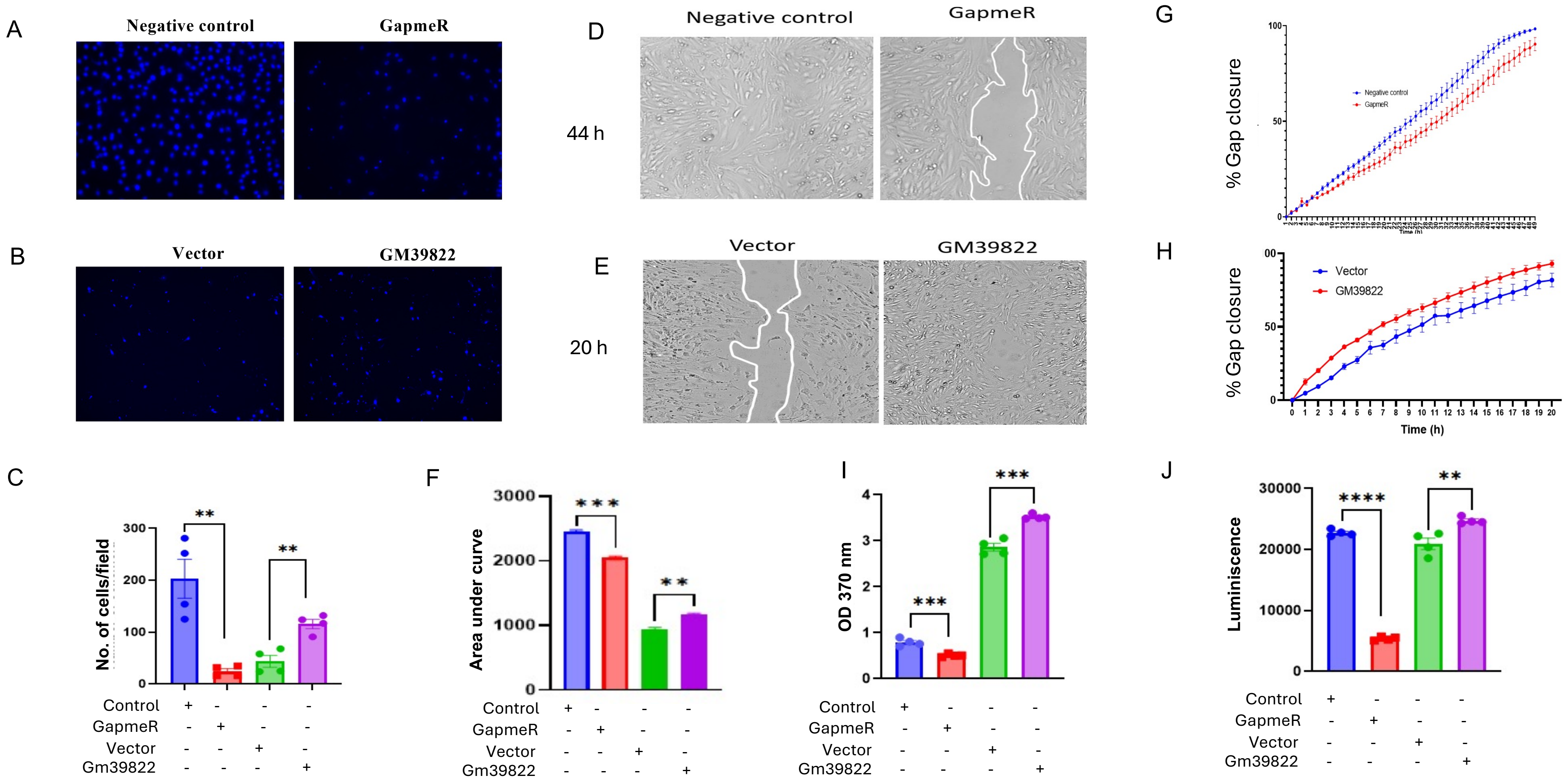

Supplementary Figure S6. Gm39822 regulates migration and proliferation of bEND.3 cells

(A-B) Representative images of transwell migration of bEND.3 cells after (A) knockdown and (B) overexpression of Gm39822; (C) quantification of the cells/field, n=4/group.

(D-H) Representative images of scratch assay, area under curve and % gap closure after (D, G, and F) knockdown and (E, H, and F) overexpression of Gm39822, n=4/group.

(I) Proliferation of bEND.3 cells in response to Gm39822 knockdown or overexpression, n=4/group.

(J) Caspase 3/7 activity in bEND.3 cells after Gm39822 knockdown or overexpression, n=4/group. The data is represented as mean ± SEM and statistical significance was determined by unpaired two-tailed Student's t test. *p < 0.05, **p < 0.01, ***p < 0.001, ****p < 0.0001..
