# Supplementary material for: Endothelial-Enriched lncRNA Gm39822 Modulates Inflammation and Dysfunction in Non-Diabetic Endothelial Cells
Source: Int J Mol Sci. 2025 Aug 22;26(17):8147. doi: 10.3390/ijms26178147 (PMC12427768; doi:10.3390/ijms26178147)
Supplement: Supplementary file 1 [file ijms-26-08147-s001.zip › 1-ijms-3810727-supplement-8.22/Table S1_MWF.pdf]

Table S1

	Gene	**Forward primer (5'->3')**	**Reverse primer (5'->3')**
1	*Gm9822*	AACCTTGGATGGTGGCGTTT	ATGCAAAGGCTCAAAAATGGTT
2	*Il-6*	CTGCAAGAGACTTCCATCCAG	AGTGGTATAGACAGGTCTGTTGG
3	*Vcam-1*	AGCTGGAACGAAGTATCCACG	GGAGCCAAACACTTGACCGT
